# Pesticide Application among Farmers in the Catchment of Ashaiman Irrigation Scheme of Ghana: Health Implications

**DOI:** 10.1155/2015/547272

**Published:** 2015-12-21

**Authors:** Memuna M. Mattah, Precious A. D. Mattah, Godfred Futagbi

**Affiliations:** ^1^Department of Environmental and Development Studies, Central University College, Accra, Ghana; ^2^Directorate of Academic Planning and Quality Assurance, University of Cape Coast, Cape Coast, Ghana; ^3^Department of Animal Biology and Conservation Science, University of Ghana, Legon, Accra, Ghana

## Abstract

Pesticide use in modern day agriculture has increased tremendously. Pesticides are used to control pests and weeds, as well as protect crops from postharvest losses; however, their effects on humans and the environment cannot be overstated. This study examined pesticide acquisition, handling, and use among 120 farmers within the catchment of a small urban irrigation scheme. Also, in-depth interviews and focus group discussions were conducted among selected farmers through which further data was collected to augment that of the survey. Twelve types of pesticides, including herbicides, insecticides, and fungicides, were found in use in the study areas. Three main sources of information about pesticides were identified, 43.3% from extension officers, 39.2% from agrochemical dealers, and 10% from colleague farmers. Seventy-five percent (75%) of the respondents purchased the pesticides from agrochemical shops. Out of 74 farmers who were observed spraying pesticides on their farms, only 25.7% wore dresses that covered their whole body but without goggles. About sixty-seven percent (66.7%) of the farmers whose chemical got finished left the containers on their farms or threw them into the bushes around. The frequency of application was influenced by affordability and size of farm, among others. The study recommended that training of farmers on pesticide handling and use should be intensified.

## 1. Introduction

Agriculture remains the main economic stay of Ghana. It employs over 50% of the labour force and contributes above 20% to the GDP [1, 2]. In spite of this, agricultural practices in Ghana remain rudimentary resulting in low yields and productivity [3]. As a way of curbing low yields and productivity, farmers resort to the use of pesticides. Pesticide use among farmers in Ghana has reached its peak in recent years especially for controlling weeds, pests, and preservation of harvested crops [3]. In Ghana, pesticides are applied mainly to cash crops and vegetables [4]. While many farmers lack adequate information about the hazards associated with handling and use of pesticides, several reports discussed the effects of pesticides on the environment and on the health of farmers [5–8].

The government of Ghana in mid-1960s constructed an irrigation dam in Ashaiman, a fast developing city in the Greater Accra region of Ghana. The dam has since its construction helped farmers in Ashaiman and its environs in the cultivation of various crops especially vegetables and rice for the urban market. It is therefore important that the incessant use of agricultural pesticides among the farmers be constantly investigated in order to assess the possible public and environmental health risk that may be associated with their use. This paper is aimed at evaluating the complete chain from information on pesticides, mode of acquisition, handling, use, and disposal of waste containers among farmers in the environs of Ashaiman in Ghana. It was part of a major study on the effect of urbanization on water quality in the Ashaiman Irrigation Scheme.

## 2. Methods

### 2.1. Study Area

**Figure 1:**
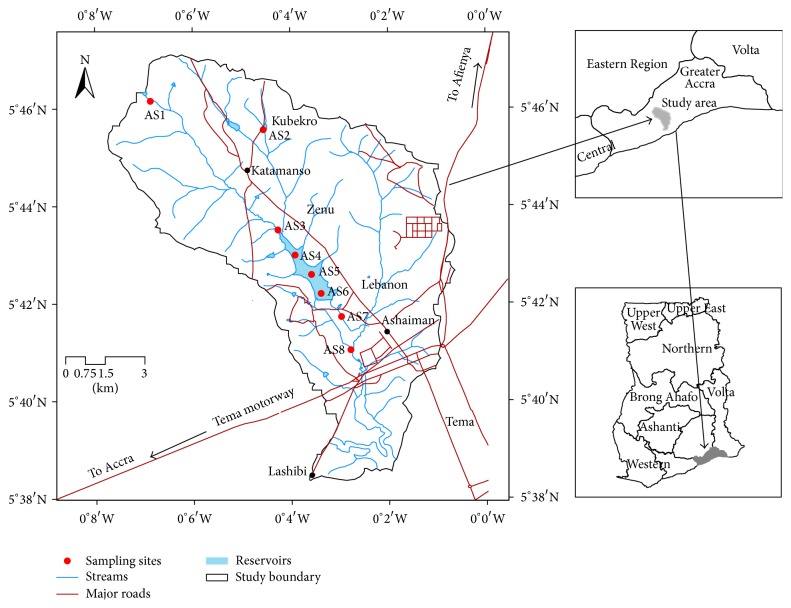
Map of the study area.

The survey was conducted in four communities found within the catchment of Dzorwulu stream which was dammed into the Ashaiman irrigation scheme. These communities include Katamanso, Kubekro, Zenu, and Lebanon (Figure 1). While the first-three communities are located north of the irrigation scheme, Lebanon, on the other hand, shares boundary with the irrigated lands and for that matter has most farmers who work on the irrigated fields. The stream was dammed between Zenu and Lebanon and drained into the sea through the Sakumo Lagoon. Farmers cultivate different types of crops such as okra, cabbage, pepper, lettuce, maize, and rice for the urban markets. Crops such as cassava and maize are mainly cultivated at the upstream of the irrigation scheme.

### 2.2. Data Collection

A survey instrument was developed, pretested, and administered to 120 households which were involved in farming activities within the catchment of the stream. The distribution of households selected for the survey was 25 each from Katamanso and Kubekro, 30 from Zenu, and 40 from Lebanon based upon population of farming households in the communities. Households were randomly sampled from houses that had been selected systematically through the sampling of every 5th house in each community starting from the northernmost house of the community using a handheld Garmin Global Positioning System (GPS) navigator (Garmin Inc., Kansas, USA). Final selection of households was based on engagement in crop cultivation. The survey instrument solicited information on ownership of farm(s), types of crops cultivated, sources of information on pesticides, sources of pesticides, knowledge on pesticides, and frequency of use of pesticides among others. Two focus group discussions (FGDs) were organized for further information to augment what was gathered through the survey. Selected farmers from the two upstream communities, Kubekro and Katamanso, were organized for the first FGD. The second FGD was organized for Zenu and Lebanon which were in the midstream and downstream, respectively. Observations were also made on use of protective clothes during spraying of pesticides and on the disposal of pesticides containers after use. The importance of the survey was explained to the farmers and their consent was sought before its administration.

### 2.3. Data Analysis

The Statistical Package for Social Sciences (SPSS) version 16 (SPSS Inc., Chicago, IL) was used in capturing, cleaning, and analyzing the data. All charts and tables were drawn using Microsoft excel 2007 and GraphPad Prism (GraphPad Prism, GraphPad Software, San Diego, CA, USA). Using content analysis techniques, various themes which espoused the views of farmers were derived from the FGDs.

## 3. Results

### 3.1. Background of Farmers

The background characteristics of the 120 farmers who responded to the survey are presented in Table 1. Forty percent and 48% of the respondents from Kubekro and Katamanso, respectively, were females and 45% of all respondents were females. At least over 50% of all respondents had up to secondary education while about 22% had no education. With regard to age distribution, approximately 13% were below 20 and most (over 70%) were between 20 and 60 years of age.

### 3.2. Ownership of Fields and Farming Practices among Respondents

**Figure 2:**
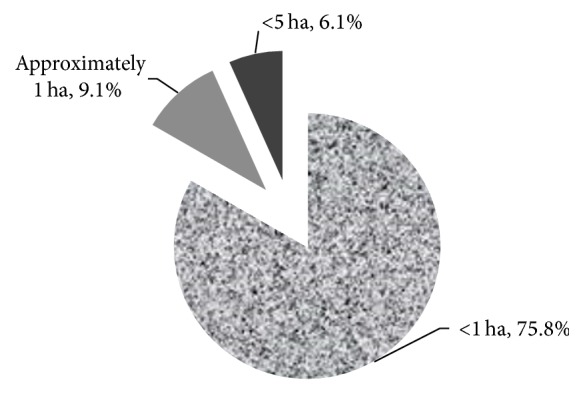
Average size of fields owned by respondents.

Eighty percent (80%) of respondents had only one, while 15% had two and 5% had three or more fields on which they farmed in the scheme area. Size of the farms varies with 76% below one hectare, 9% of approximately one hectare, and 6% between one and five hectares (Figure 2). Forty-three (43%) percent of the respondents owned the fields they cultivated while 82.4% of those who did not own the fields cultivated them on leasehold and the rest cultivated them on sharecropping basis.

### 3.3. Types of Crops Cultivated by the Farmers and Cultural Practices

Types of crops grown by farmers include maize, cassava, vegetables, rice, and fruits. Fifty percent (50%) of the respondents cultivated maize, 29% vegetables, 12.5% rice, and the rest cassava and fruits. Maize, cassava, and fruits were mainly grown in the upstream of the scheme, that is, in Katamanso and Kubekro, while vegetables were cultivated in midstream and downstream, in Zenu and Lebanon. Rice was mainly cultivated in the downstream of the scheme at Lebanon area. Tomatoes, okra, and pepper were the main vegetables and watermelon was the main fruit grown in the study areas. Additionally, apart from maize which was cultivated on all sizes of farm, the rest of the crops were grown on less than one-hectare farmland (Table 2).

Herbicides were mainly used by 41.7% of the respondents to clear the lands of weeds for planting of crops, while 24.2% used slash and burn to control the weeds. About twenty-four percent (24.2%) of the respondents used machines to plough the weeds with the soil, that is, if the weeds were not too grown. Results show that 66.7% and 62.5% of farmers in Zenu and Lebanon, respectively, were the main user of herbicides. On the other hand, majority (36.4%) of the farmers who used machines to plough the weeds were from Kubekro. Rudimentary instruments such as hoes and cutlasses were the most used by 84.2% of the respondents for turning the soil. Almost 59% of the respondents engaged in monocropping and 41.4% did crop rotation.

### 3.4. Pesticides In-Use in Ashaiman

Twelve different types of pesticides were in-use in Ashaiman and its environs. Thirty-five percent of the respondents used round-up (glyphosate 41%), while 8.3% used gramoxone (paraquat 276 g/L) for controlling weeds. Also 11.7% and 10% used karate (Lambda-cyhalothrin 25 g/L) and dimethoate (dimethoate 400 g/L), respectively, for controlling insect pests. Topcope (sulphur 50% and copper sulphate 8.4%) was the only fungicide in-use in the study areas. In exception of Master (Bifenthrin), all the pesticides were used by respondents in Lebanon suburb of Ashaiman where the irrigation fields are found. All those who used Dursban (Chlorpyrifos-ethyl 480 g/L) were in Lebanon. Five of the pesticides were used in Zenu and these include karate (Lambda-cyhalothrin 25 g/L), round-up (glyphosate 41%), Stam F34 (Propanil 360 g/L), Pawa (Lambda-cyhalothrin 25 g/L), and gramoxone (paraquat 276 g/L). Only four of the pesticides were used in Kubekro (Topcope, Master, karate, and Cydim super EC) and Katamanso (Cydim super EC, gramoxone, dimethoate, and Master). Additionally, karate was used in all the communities (Table 3).

The pesticides were grouped into insecticides, herbicides, and fungicides. Insecticides and herbicides were mostly used compared to the fungicides. With regard to WHO/FAO classification, most of the pesticides belong to category II of moderately hazardous chemicals (7/12), out of which 6 were insecticides. Indeed all synthetic insecticides belong to category II (Table 4).

### 3.5. Pesticide Application

Seventy percent (70%) of the farmers had been using pesticides for over 5 years whereas only 8.3% did not use synthetic pesticides at all in their farming activities. Those who did not use the synthetic pesticides rather used natural repellants such as neem (*Azadirachta indica*) seed extracts to control pests. Though 80% of the respondents stated that the pesticides were readily available on sale in Ashaiman, only 35.8% found the pesticides to be affordable and as much as 51% found them to be too expensive.

**Figure 3:**
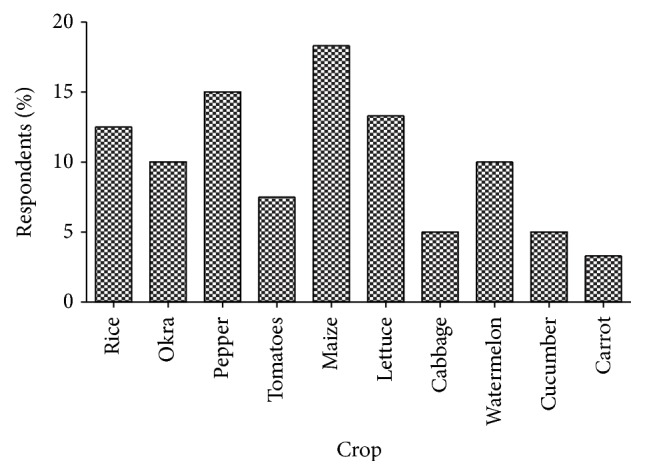
Crops to which pesticides were applied.

The pesticides were applied to prepare the lands and to control pests of various crops (Figure 3). Over 18% of the farmers applied pesticides to maize farms, 15% to pepper, and 13% to lettuce. In the focus group discussions, the discussants mentioned that the frequency of application of pesticides depended first and foremost on farmer's ability to purchase the pesticide, followed by type of weed (if it is herbicide), type of crop, size of farm, and type of pests attacking the crops, in that order. Majority (34.2%) of the respondents applied the pesticides twice while 28.3% applied them three times in a farming season. Another 28.3% applied them four or more times depending on the extent of infestation by pests. The data show that those who applied the pesticides more than three times cultivate less than one-hectare farms (Table 5). Quantity pesticides used among farmers ranged from 50 mL to 2000 mL per application. The first application of pesticides to crops by most respondents (88.3%) occurs between 2nd and 3rd weeks of planting. A second application is done, if it is necessary, normally in the 6th and 7th weeks as stated by 32% of the respondents. Other applications occurred as and when the farmers felt it was needed. It is worth noting that 5th and subsequent applications were done for vegetables (Table 6).

**Figure 4:**
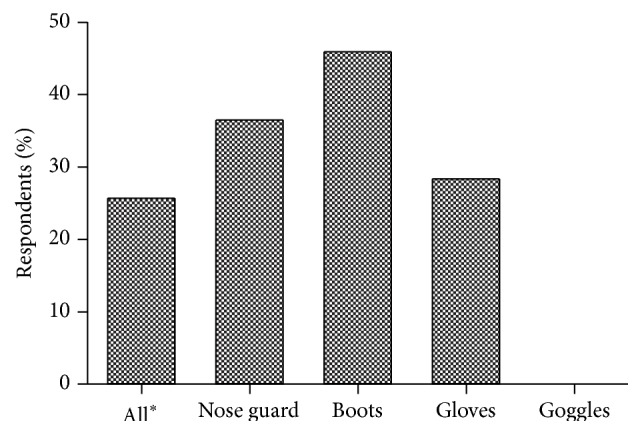
Personal protective equipment.  ^*∗*^All gears except goggles.

Out of 74 farmers who were observed spraying pesticides on their farms, 36.5% used nose guards, 45.9% used boots, 31% used hand gloves, and 25.7% wore dresses that covered the whole body except the eyes (Figure 4). Farmers were observed mixing different types of pesticides for spraying. The “cocktail” of pesticides, they believed, had increased potency for fast control of pests. The FGDs revealed that most farmers did not read and for that matter did not adhere to the instructions on how to apply the pesticides. They however received the information on the quantity of pesticides to use and how to apply them from the vendors, colleague farmers, and extension officers. Field observations showed that empty pesticide containers were thrown into nearby bushes after use. At least 16 out of 24 (66.7%) of those whose chemicals got finished left the containers on their farm or threw them into the nearby bushes. On how the chemicals were preserved, most (86%) farmers acknowledged keeping the chemicals in their homes and not on the field for fear of theft.

### 3.6. Sources of Information and Acquisition of Pesticide

Information on particular pesticides to use on crops was obtained from three sources. About forty-three percent (43.3%) of the respondents had information about various pesticides through training programmes organized by agricultural extension officers. Also, 39.2% of the respondents had information from agrochemical dealers in Ashaiman and its environs while 10% of them received theirs from colleague farmers. Most (75%) of the respondents purchased the pesticides from agrochemical shops while 10.8% acquired theirs from other sources, such as friends and relatives.

## 4. Discussion

The importance of pesticides in modern agriculture cannot be overemphasized; however, use of pesticides comes with concerns regarding public and farmers' health as well as environmental pollution. Health problems associated with pesticide use, especially in farmers in developing countries, are well documented [10, 11]. The data indicate that most of the farmers had attained levels of education that should enable them to read, in spite of the fact that many of them do not read the labels and instructions on pesticides before using them. They rather depend on the recommendation from chemical dealers, extension officers, colleagues, and sometimes their own intuition on how to apply the pesticides. The reason for this is not clear but it has been shown that farmers prefer to rely on pesticide sellers, extension officers, and peer farmers rather than reading instructions on or flyers attached to the chemicals [12]. The attitude of farmers in this study is similar to that studied by Alam and Wolff [12]. Therefore, equipping pesticide sellers with relevant and adequate information and involving them in farmer education on pesticide use will greatly complement the work of the extension workers [12].

Lack of adequate knowledge about pesticide use had reflected in the poor handling, frequency, and timing of application of the chemicals with serious health consequences. The farmers mentioned that the frequency of application of pesticides depends first and foremost on farmer's ability to purchase the pesticide, followed by type of weed, type of crop, size of farm, and type of pests attacking the crops, in that order. In spite of the above the quantity of pesticides used per application among respondents ranged from 50 mL to 2000 mL, indicating that there could be underdoses or overdoses depending on the size of the field. The results show that those who applied the pesticides more than three times cultivated farms less than one hectare in size. A small farm actually favours the foremost factor that influences the frequency of application, that is, the ability to afford to pay for the pesticides. Obviously, those having small farms would like to keep productivity high as well and they do this by frequent use of chemicals [13]. This implies that access to bigger farms may be an antidote to indiscriminate use of pesticides.

Most commonly used pesticides were herbicides and insecticides with a minimal use of fungicides. Insecticide use was higher among the vegetables, maize, and rice farmers than cassava. Herbicide use was high among rice farmers and the reason for that is high cost and nonavailability of farm labour in the urban areas. The use of protective clothes among farmers was low as reported in other parts of the country and elsewhere [7, 8]. The results show that most of the pesticides and all the synthetic insecticides were category II chemicals. Though in applying categories I and II chemicals farmers were supposed to wear appropriate personal protective equipment that covers the entire body [14], only 26% of the farmers covered their body except the eyes when applying the pesticides. Additionally, less than 50% of the farmers used either of the protective equipment items such as nose guards, boots, and gloves while none used goggles. This shows that the farmers had dangerously exposed themselves to the toxic chemicals. Some of the pesticides, for example, organophosphate pesticides such as dursban, are known to have negative consequence on proper functioning of the nervous system [15, 16]. Additionally, it is emerging that glyphosate (the active ingredient in round-up, the most commonly used pesticide in the study area), which was thought to be not harmful to humans and animals, is actually harmful. Among others, glyphosate is now associated with cancer and known to destroy beneficial bacteria in humans, thereby allowing pathogenic ones to overgrow and overwhelm the body [17, 18].

The data also indicate the need to educate farmers on the mode of storage of pesticides and disposal of pesticide wastes. High numbers of farmers store their chemicals at home and this could lead to increase in morbidity or injury rate among farmers' families. The disposal methods are of equal concern since these can affect the larger community resulting from leaching of the pesticides into water bodies, which can lead to accidental ingestion.

## 5. Conclusions

In order to maximize output on the limited land, farmers are bent on practicing modern agricultural methods including the use of pesticides to protect their crops from pest damage. There is therefore the need to increase the number of extension officers to educate farmers on the right use of pesticides, and equipping pesticide sellers with relevant information and involving them in farmer education on pesticide use will complement the work of the extension workers. It would be prudent for the Environmental Protection Agency to monitor the proper disposal of pesticide containers to avert poisoning and environmental pollution.

## Figures and Tables

**Table 1:** Farmer's personal information.

Characteristics	Variable (%)	Community			
		Kubekro	Katamanso	Zenu	Lebanon
Gender	Male	60.0	52.0	56.7	52.5
	Female	40.0	48.0	43.3	47.5

Educational status	None	16.0	32.0	20.0	20.0
	Primary	16.0	28.0	40.0	30.0
	Middle/JSS	32.0	16.0	40.0	20.0
	Secondary	24.0	8.0	16.0	17.5
	Postsecondary	8.0	16.0	4.0	7.5
	Tertiary	4.0	0.0	0.0	5.0

Age group	Below 20	8.0	16.0	16.7	12.5
	20–29	28.0	16.0	16.7	20.0
	30–39	32.0	20.0	20.0	20.0
	40–49	20.0	20.0	26.7	17.5
	50–59	8.0	16.0	6.7	12.5
	60+	4.0	12.0	13.3	17.5
*N*	120	25	25	30	40

**Table 2:** Type of crop and size of farm.

Crop	Size of farm			Total
	<1 ha	1 ha	>5 ha	
Maize	39	11	8	58
Rice	15	0	0	15
Vegetables/fruits	40	0	0	40
Cassava	7	0	0	7

**Table 3:** Type of pesticides in-use at various farm sites.

Pesticides	Percentages				
	Respondents	Lebanon	Kubekro	Katamanso	Zenu
Cydim super EC	5.8	0.8	3.3	1.7	0.0
Round up	35.0	60.0	0.0	0.0	8.0
Gramoxone	8.3	5.0	0.0	0.0	22.2
Pawa	3.3	2.5	0.0	0.0	8.3
Dimethoate	10.0	10.0	9.5	26.1	0.0
Dursban	0.8	2.5	0.0	0.0	0.0
Karate	11.7	7.5	14.3	21.7	8.3
Master	4.2	0.0	4.8	17.4	0.0
Stam F34	1.7	2.5	0.0	0.0	2.8
Topcope	0.8	2.5	0.0	0.0	0.0
Chemosate	0.8	2.5	0.0	0.0	0.0

**Table 4:** Active ingredients in the pesticides used by farmers and WHO/FAO classification.

Types of pesticides	Common name	Active ingredients	WHO/FAO classification
Herbicides	Stam F 34	Propanil 360 g/L	III
	Round-up	Glyphosate 41%	III
	Gramoxone	Paraquat 276 g/L	II
	Chemosate	Glyphosate 41%	III

Insecticides	Dimethoate	Dimethoate 400 g/L	II
	Dursban 4E	Chlorpyrifos-ethyl 480 g/L	II
	Pawa 2.5 EC	Lambda-cyhalothrin 25 g/L	II
	Cydim super	Dimethoate 400 g and cypermethrin 36 g	II
	Karate 2.5 EC	Lambda-cyhalothrin 25 g/L	II
	Master	Bifenthrin	II
	Neem seed extracts	Azadirachtin	U

Fungicides	Topcope	Sulphur 50% and copper sulphate 8.4%	III

*Note.* II = moderately hazardous; III = slightly hazardous; U = unlikely to present acute hazard in normal use [9].

**Table 5:** Size of farm and frequency of application.

Frequency of application	Size of farm			Total
	<1 ha	1 ha	>5 ha	
Once	9	0	0	9
Twice	25	4	5	34
Three times	22	3	3	28
Four times	15	0	0	15
5–10 times	7	0	0	7
More than 10 times	4	0	0	4
Not applicable^*∗*^	19	4	0	23
Total	101	11	8	120

^*∗*^Not applicable refers to farmers who used neem seed extracts.

**Table 6:** Type of crop and frequency of application of pesticides.

Frequency of application	Crops				Total
	Vegetables	Maize	Rice	Cassava	
Once	2	3	3	1	9
Twice	8	16	5	6	35
Three times	7	19	4	0	30
Four times	7	5	0	0	12
5–10 times	6	0	0	0	6
More than 10 times	5	0	0	0	5
Not applicable^*∗*^	5	15	3	0	23
Total	40	58	15	7	120

^*∗*^Not applicable refers to farmers who used neem seed extracts.
